# Correction: *Prepayment meters strongly associated with multiple types of deprivation and emergency respiratory hospital admissions: an observational, cross-sectional study*

**DOI:** 10.1136/jech-2023-220793corr1

**Published:** 2024-03-08

Ding X, Akimova ET, Zhao B, *et al*. Prepayment meters strongly associated with multiple types of deprivation and emergency respiratory hospital admissions: an observational, cross-sectional study. *J Epidemiol Community Health* 2024;78:54-60. doi: 10.1136/jech-2023-220793

